# Disorder‐Enabled Directional Delocalization and Wave Steering in Time‐Modulated Dirac Materials

**DOI:** 10.1002/nap2.70103

**Published:** 2026-04-23

**Authors:** Seulong Kim, Kihong Kim

**Affiliations:** ^1^ Research Institute of Basic Sciences Ajou University Suwon Korea; ^2^ Department of Physics Ajou University Suwon Korea; ^3^ School of Physics Korea Institute for Advanced Study Seoul Korea

## Abstract

We demonstrate a disorder‐enabled yet localization‐immune directional transport channel in time‐modulated Dirac systems subject to stochastic temporal variations of a vector potential. In a spatially uniform medium, random temporal modulation induces strong Anderson localization for generic propagation directions, whereas waves propagating parallel to the modulation axis remain perfectly delocalized. This behavior originates from the pseudospin structure of the Dirac equation, which enforces exact suppression of interband coupling for specific propagation directions, thereby eliminating disorder‐induced backscattering. As a result, temporal disorder acts as a symmetry‐selective angular filter, producing highly collimated transport without spatial structuring. Unlike conventional impedance matching–based transmission in clean time‐varying media, this mechanism arises intrinsically from stochastic temporal modulation and remains robust across a wide range of disorder models. These findings establish temporal disorder as a resource for direction‐selective wave control, enabling reconfigurable beam steering, adaptive filtering, and disorder‐tolerant nanophotonic components such as temporal beam shapers. More broadly, this phenomenon represents a temporal analogue of disorder‐induced delocalization channels known in spatially disordered systems and demonstrates that randomness, typically associated with localization and transport suppression, can instead isolate a perfectly transmitting channel through symmetry‐selective dynamics.

## Graphical abstract

Random temporal modulation of a vector potential in a Dirac medium produces disorder‐enabled directional delocalization: waves propagating parallel to the modulation direction remain perfectly delocalized, whereas those in all other directions undergo strong Anderson localization. Temporal disorder thus acts as a directional filter, enabling collimated and reconfigurable wave transport without spatial structuring.

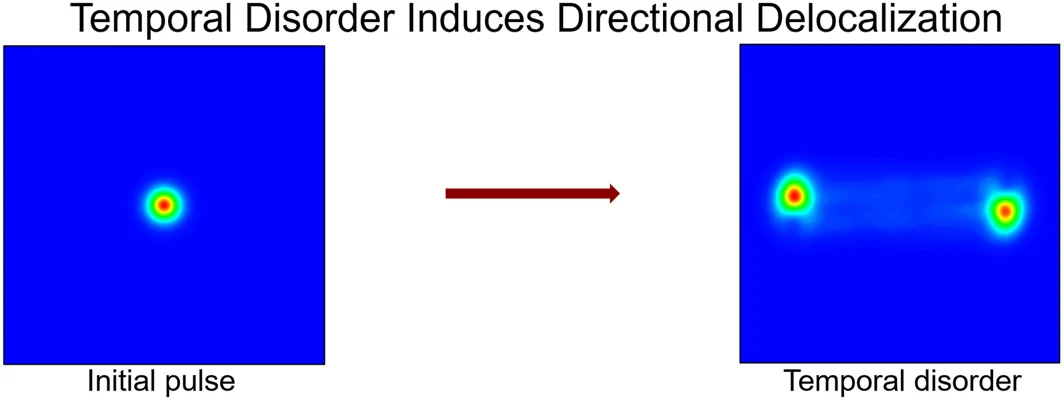

## 1 Introduction

Dynamic control of wave transport in nanophotonic and electronic systems underpins key functionalities such as beam steering, routing, filtering, and adaptive signal processing. Recent advances in time‐varying media have revealed a wide range of unconventional phenomena, including temporal reflection and refraction, photonic time crystals, and momentum gaps in periodically modulated systems [1, 2, 3, 4, 5, 6, 7, 8, 9, 10, 11, 12, 13, 14, 15, 16]. In parallel, stochastic temporal modulation has been shown to induce Anderson localization through disorder‐driven interference, even without spatial inhomogeneity [17, 18, 19, 20, 21, 22, 23]. Together, these developments have significantly broadened the scope of wave manipulation beyond static material responses.

In clean time‐modulated systems, exact temporal impedance‐matching conditions can eliminate backward scattering at temporal interfaces, enabling reflectionless transmission for specific parameter configurations [24]. In Dirac systems, related matching conditions arise naturally from the pseudospin structure and can be derived analytically [25, 26]. When temporal modulation is instead stochastic, wave dynamics are typically dominated by localization effects, suppressing transport for most propagation directions. Whether disorder can instead enable robust, direction‐selective transport channels in time‐modulated systems remains an open question.

To place our results in context, it is important to distinguish two related but fundamentally different phenomena. The temporal Brewster effect is a deterministic mechanism: Exact impedance matching conditions at a temporal interface yield perfect transmission for specific parameter configurations, in direct analogy with spatial Brewster transmission at a planar interface [24]. It does not rely on disorder and is absent in stochastic settings. The Brewster anomaly, by contrast, is intrinsically disorder‐enabled [27, 28, 29, 30]. Originally identified in randomly stratified spatial media, it describes the survival of a perfectly transmitting channel under strong disorder, whereas all other channels undergo Anderson localization. The two phenomena are therefore distinct in both origin and mechanism and should not be conflated.

Here, we demonstrate that time‐modulated Dirac systems support a directional transport channel that is both disorder‐enabled and immune to localization, representing a temporal analogue of disorder‐induced delocalization channels associated with the Brewster anomaly. Although random temporal fluctuations induce strong Anderson localization for generic propagation directions, waves propagating parallel to the modulation axis remain perfectly delocalized under strong disorder. This behavior originates from the pseudospin structure of the Dirac equation, which enforces exact suppression of interband coupling for specific propagation directions, thereby eliminating disorder‐induced backscattering. Temporal disorder thus acts as a symmetry‐selective angular filter that isolates a single perfectly transmitting channel, producing highly collimated transport without any spatial structuring. The propagation direction can be dynamically controlled through the modulation axis, enabling purely temporal steering of wave transport.

Related studies have reported disorder‐enabled, direction‐selective suppression of localization in spatially disordered Dirac systems [31, 32, 33], as well as Brewster‐type total transmission in layered media with long‐range randomness and in temporally periodic systems under impedance matching conditions [34, 35]. Although the present mechanism shares the disorder‐enabled delocalization character of the spatial Brewster anomaly, it is rooted in pseudospin‐selective channel decoupling under temporal modulation rather than polarization‐dependent impedance matching at spatial interfaces. It therefore represents a distinct class of symmetry‐protected, disorder‐enabled transport in which randomness, typically associated with localization and suppression, acts as a filter that isolates a perfectly transmitting channel through symmetry‐selective dynamics.

Beyond its fundamental significance, this mechanism enables dynamic beam steering governed entirely by temporal modulation. In electronic Dirac materials, a time‐dependent vector potential can be tuned via external electric fields through E(t)=−∂A(t)/∂t, while in photonic Dirac lattices, the same effect can be emulated through synthetic gauge‐field modulation. These results establish temporal disorder as a practical design resource for reconfigurable, disorder‐tolerant control of wave propagation in both photonic and electronic Dirac platforms, with direct implications for temporal beam shapers, adaptive filters, and related nanophotonic components. This identifies a new class of disorder‐enabled transport in time‐modulated systems, in which localization is not universally suppressive but instead selectively eliminates all but a symmetry‐protected propagation channel.

## 2 Models

We investigate wave dynamics governed by the massless pseudospin‐1/2 Dirac equation in a spatially uniform medium subject to a time‐dependent vector potential, $A\left(t\right)=A\left(t\right)\hat{x}$, aligned along the x‐axis with a temporally varying amplitude. The wave vector k forms an angle θ with the x‐axis, which serves as the key geometric parameter characterizing the propagation direction. Spatial uniformity of the medium ensures that k remains constant throughout the temporal evolution, so that θ is fixed when the modulation direction is fixed; more generally, the physically relevant parameter is the angle between k and the instantaneous direction of A(t). Gauge invariance of the temporal scattering process is discussed in Supporting Information S1: Section S1.

The time‐dependent Dirac equation for the two‐component spinor $\Psi ={\left(\psi_{1},\psi_{2}\right)}^{T}$ reads

(1) $$i\hbar \frac{d}{dt}\Psi \left(t\right)=\left(\begin{array}{cc} 0 & v_{F}\left(\pi_{x}-i\pi_{y}\right)\\ v_{F}\left(\pi_{x}+i\pi_{y}\right) & 0 \end{array}\right)\Psi \left(t\right),$$

with

(2) $$\pi_{x}=\hbar k\;\cos\;\theta +eA\left(t\right),\;\pi_{y}=\hbar k\;\sin\;\theta ,$$

where k=|k|, e is the electron charge, and $v_{F}$ is the Fermi velocity.

Eliminating $\psi_{2}$, the equation reduces to

(3) $$\frac{d}{dt}\left[\frac{1}{\varepsilon \left(t\right)}\frac{d\psi_{1}}{dt}\right]+\omega_{0}^{2}\varepsilon^{*}\left(t\right)\psi_{1}=0,$$

where

(4) $$\varepsilon \left(t\right)=e^{-i\theta }+\alpha \left(t\right),\;\alpha \left(t\right)=\frac{eA\left(t\right)}{\hbar k},\;\omega_{0}=kv_{F}.$$

Where ε(t) is a complex parameter unrelated to electromagnetic permittivity. The corresponding group velocity is

(5) $$v_{g}=\pm v_{F}\left(\frac{\alpha +\cos\;\theta }{\left|\varepsilon \right|},\frac{\sin\;\theta }{\left|\varepsilon \right|}\right),$$

where ± corresponds to particle‐like (p) and hole‐like (h) branches. In general, the vector potential renders the group and phase velocities noncollinear, except for propagation along θ=0 or π.

We model the vector potential as

(6) $$\alpha \left(t\right)=\alpha_{0}+\delta \alpha \left(t\right),$$

where δα(t) represents stochastic temporal fluctuations. To demonstrate robustness, we consider two complementary models. In Model 1, δα(t) is Gaussian white noise with

(7) $$\langle \delta \alpha \left(t\right)\delta \alpha \left(t^{\prime }\right)\rangle =g_{0}\delta \left(t-t^{\prime }\right),\;\langle \delta \alpha \left(t\right)\rangle =0,$$

which allows analytical treatment via invariant imbedding. In Model 2, δα(t) is piecewise constant over intervals of duration $\tau_{d}$, taking independent values uniformly distributed in [−Δα,Δα]. Wave propagation is obtained by averaging over many realizations. The agreement between these models demonstrates that the disorder‐enabled directional delocalization is robust and independent of the specific statistical form of temporal disorder.

## 3 Methods

### 3.1 Invariant Imbedding Formulation

We summarize the invariant imbedding formulation used for numerical analysis. We consider an incident particle‐like (p) wave of unit amplitude, $\psi_{1}\left(t\right)=e^{-i\omega_{1}t}$, for t≤0, with spatial dependence $e^{i\left(k_{x}x+k_{y}y\right)}$. When the vector potential varies within the interval 0≤t≤T, the wave function takes the form

(8) $$\psi_{1}\left(t,T\right)=\left\{\begin{array}{cc} e^{-i\omega_{1}t}, & t<0,\\ r\left(T\right)e^{i\omega_{2}\left(t-T\right)}+s\left(T\right)e^{-i\omega_{2}\left(t-T\right)}, & t>T, \end{array}\right.$$

where r(T) and s(T) are the temporal reflection and transmission coefficients, respectively. The frequencies $\omega_{i}$ (i=1,2) satisfy

(9) $$\frac{\omega_{i}}{\omega_{0}}=|\varepsilon_{i}|=\sqrt{1+\alpha_{i}^{2}+2\alpha_{i}\;\cos\;\theta },$$

with $\alpha_{1}$ and $\alpha_{2}$ as the initial and final normalized vector potentials, respectively.

The coefficients r and s describe interband (p→h) and intraband (p→p) transitions, respectively. Using invariant imbedding [36, 37, 38, 39, 40, 41, 42], their evolution with respect to the modulation duration τ is governed by

(10) $$\frac{1}{\omega_{0}}\frac{dr}{d\tau }=i\beta r+\gamma s,\;\frac{1}{\omega_{0}}\frac{ds}{d\tau }=-\gamma r-i\beta s,$$

where

(11) $$\beta =\frac{1+\alpha \alpha_{2}+\left(\alpha +\alpha_{2}\right)\cos\;\theta }{\left|\varepsilon_{2}\right|},\;\gamma =\frac{\left(\alpha -\alpha_{2}\right)\sin\;\theta }{\left|\varepsilon_{2}\right|}$$

The initial conditions are

(12) $$r\left(0\right)=\frac{1}{2}\left(1-\frac{\varepsilon_{2}}{\varepsilon_{1}}\frac{\left|\varepsilon_{1}\right|}{\left|\varepsilon_{2}\right|}\right),\;s\left(0\right)=\frac{1}{2}\left(1+\frac{\varepsilon_{2}}{\varepsilon_{1}}\frac{\left|\varepsilon_{1}\right|}{\left|\varepsilon_{2}\right|}\right)$$

and the reflectance and transmittance

(13) $$R=|r{|}^{2},\;S=|s{|}^{2}$$

satisfy the conservation law

(14) R+S=1.

Detailed derivations are provided in Supporting Information S1: Section S2–S4.

Equation (10) has the structure of a two‐level system, where β governs intraband phase accumulation and γ controls interband (pseudospin) coupling. Because γ∝sinθ, the coupling vanishes identically at θ=0 and π, leading to an exact decoupling of pseudospin channels. As a result, temporal reflection is suppressed and the dynamics remain immune to arbitrarily strong temporal disorder along these directions.

### 3.2 Moments of Reflection and Transmission Coefficients

To characterize long‐time transport under stochastic temporal modulation, we analyze disorder‐averaged moments of the reflection and transmission coefficients. Instead of tracking individual realizations of r and s, we derive closed evolution equations for their statistical moments $Z_{\mathrm{abcd}}$. The resulting hierarchy follows directly from the linear coupling in Equation (10) combined with Gaussian stochastic averaging.

Applying the Furutsu–Novikov formula to Equation (10) [43, 44], we obtain a coupled system of differential equations for the moments $Z_{\mathrm{abcd}}=\langle r^{a}{\left(r^{*}\right)}^{b}s^{c}{\left(s^{*}\right)}^{d}\rangle$ as follows:

(15) $$\begin{array}{ll} & \frac{1}{\omega_{0}}\frac{d}{d\tau }Z_{\mathrm{abcd}}=C_{1}Z_{\mathrm{abcd}}\;+C_{2}Z_{a+1,b,c-1,d}\\ & +C_{3}Z_{a-1,b,c+1,d}\;+C_{4}Z_{a,b+1,c,d-1}\\ & +C_{5}Z_{a,b-1,c,d+1}+C_{6}Z_{a+1,b+1,c-1,d-1}\\ & +\;C_{7}Z_{a-1,b-1,c+1,d+1}+C_{8}Z_{a+1,b-1,c-1,d+1}\\ & +C_{9}Z_{a-1,b+1,c+1,d-1}\;+C_{10}Z_{a+2,b,c-2,d}\\ & +C_{11}Z_{a-2,b,c+2,d}\;+C_{12}Z_{a,b+2,c,d-2}\\ & +C_{13}Z_{a,b-2,c,d+2}. \end{array}$$

The coefficients are

(16) $$\begin{array}{ll} & C_{1}=i\left(a-b-c+d\right)\beta_{0}-\frac{g}{2}{\left(a-b-c+d\right)}^{2}\zeta^{2}\\ & \;\;\;-\frac{g}{2}\left(a+b+c+d+2ac+2bd\right)\eta^{2},\\ & C_{2}=-c\gamma_{0}-igc\left(a-b-c+d+1\right)\zeta \eta ,\\ & C_{3}=a\gamma_{0}+iga\left(a-b-c+d-1\right)\zeta \eta ,\\ & C_{4}=-d\gamma_{0}-igd\left(a-b-c+d-1\right)\zeta \eta ,\\ & C_{5}=b\gamma_{0}+igb\left(a-b-c+d+1\right)\zeta \eta ,\\ & C_{6}=\gcd\eta^{2},\;\;C_{7}=gab\eta^{2},\\ & C_{8}=-gbc\eta^{2},\;\;C_{9}=-gad\eta^{2},\\ & C_{10}=\frac{g}{2}c\left(c-1\right)\eta^{2},\;\;C_{11}=\frac{g}{2}a\left(a-1\right)\eta^{2},\\ & C_{12}=\frac{g}{2}d\left(d-1\right)\eta^{2},\;\;C_{13}=\frac{g}{2}b\left(b-1\right)\eta^{2}, \end{array}$$

where the parameters are

(17) $$\begin{array}{ll} & \beta_{0}=\frac{1+\alpha_{0}\alpha_{2}+\left(\alpha_{0}+\alpha_{2}\right)\cos\;\theta }{\left|\varepsilon_{2}\right|},\\ & \gamma_{0}=\frac{\left(\alpha_{0}-\alpha_{2}\right)\sin\;\theta }{\left|\varepsilon_{2}\right|},\;\zeta =\frac{\alpha_{2}+\cos\;\theta }{\left|\varepsilon_{2}\right|},\\ & \eta =\frac{\sin\;\theta }{\left|\varepsilon_{2}\right|},\;g=g_{0}\omega_{0}. \end{array}$$

The initial conditions are

(18) $$Z_{\mathrm{abcd}}\left(0\right)={\left[r\left(0\right)\right]}^{a}{\left[r^{*}\left(0\right)\right]}^{b}{\left[s\left(0\right)\right]}^{c}{\left[s^{*}\left(0\right)\right]}^{d}.$$

Two structural features are central. First, all inter‐moment coupling terms are proportional to sinθ (through $\gamma_{0}$ or η), so that disorder‐induced mixing vanishes identically at θ=0 and π. Second, the stochastic contributions scale with the disorder strength g, enabling systematic analysis of temporal disorder effects. As a result, the hierarchy in Equation (15) simplifies at θ=0 and π to a decoupled form with identically vanishing reflection, while for generic angles, disorder induces interband mixing and moment coupling, leading to localization. In what follows, we numerically integrate the resulting finite closed hierarchy to obtain disorder‐averaged transport as functions of the propagation angle and disorder strength.

## 4 Results

We analyze the temporal evolution of the ensemble‐averaged reflectance 〈R〉, obtained by solving Equation (15), which is valid for arbitrary disorder strength g. For clarity, we focus on the symmetric case $\alpha_{1}=\alpha_{2}=\alpha_{0}$, where the vector potentials before and after the modulation interval coincide with their mean value during modulation.

When θ=0 or π, the parameters $\gamma_{0}$ and η vanish, and Equation (15) yields 〈R〉=0 and 〈S〉=1. These directions therefore correspond to disorder‐immune propagation with perfect transmission independent of temporal fluctuations. For general θ, analytic expressions can be obtained in both the weak and strong disorder limits. In the weak disorder regime,

(19) $$\langle R\rangle =\frac{1}{2}\left[1-\exp\left(-\frac{2g\omega_{0}\sin^{2}\;\theta }{{\left|\varepsilon_{0}\right|}^{2}}t\right)\right],$$

where $|\varepsilon_{0}|=\sqrt{1+\alpha_{0}^{2}+2\alpha_{0}\;\cos\;\theta }$. In the strong disorder regime, numerical solutions yield

(20) $$\begin{array}{ll} \langle R\rangle & =\frac{1}{2}\left(1-e^{-2g\omega_{0}t}\right)\\ & \;\times \left[1-\left(1-\frac{\sin^{2}\;\theta }{{\left|\varepsilon_{0}\right|}^{2}}\right)\exp\left(-\frac{2\omega_{0}\sin^{2}\;\theta }{g}t\right)\right]. \end{array}$$

In both limits, 〈R〉 approaches 1/2 exponentially for any θ≠0,π, indicating strong temporal localization for all propagation directions except the symmetry‐protected directions θ=0 and π.

Figure 1 shows the time evolution of 〈R〉 under representative conditions. In Figure 1a, the incidence angle is fixed at θ=π/6 with $\alpha_{0}=0$, whereas the disorder strength g is varied. In Figure 1b and c, $\alpha_{0}=0.5$ is fixed and 〈R〉 is shown for several angles in the weak (g=0.01) and strong (g=100) disorder regimes, respectively. In all cases, 〈R〉 increases monotonically from zero and saturates at 1/2, except at θ=0 and π, where it remains zero due to perfect transmission. In the weak disorder regime, increasing g accelerates the approach to saturation, whereas in the strong disorder regime, the asymptotic relaxation slows as g increases.

**FIGURE 1:**
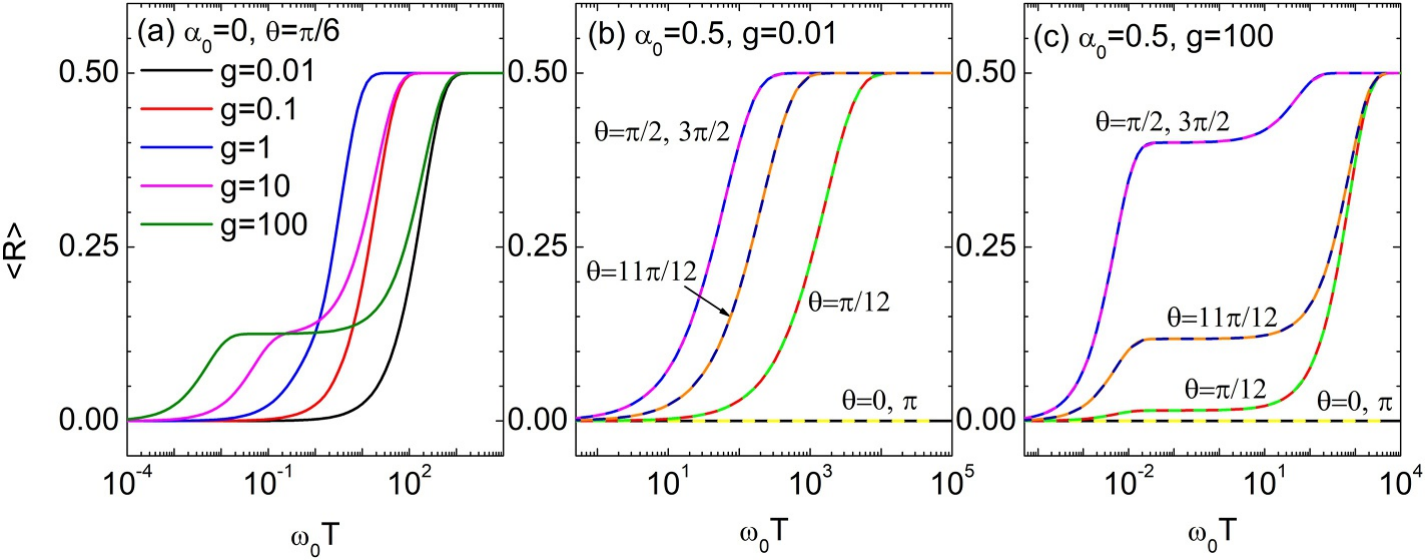
Time evolution of the disorder‐averaged reflectance 〈R〉. (a) 〈R〉 for several disorder strengths g at fixed mean vector potential $\alpha_{0}=0$ and incidence angle θ=π/6. (b) 〈R〉 for different θ in the weak disorder regime (g=0.01) with $\alpha_{0}=0.5$. (c) 〈R〉 for different θ in the strong disorder regime (g=100) with $\alpha_{0}=0.5$. Solid lines represent numerical solutions of Equation (15); dashed lines show the corresponding analytical predictions from Equation (19) in (b) and Equation (20) in (c).

Near the disorder‐immune directions $\theta_{R}=0$ or π, the reflectance exhibits universal quadratic scaling, $\langle R\rangle \propto {\left(\theta -\theta_{R}\right)}^{2}$, independent of disorder strength, mean vector potential, or modulation duration. Figure 2 confirms this scaling across representative regimes ranging from weak to very strong disorder and from short to long modulation times.

**FIGURE 2:**
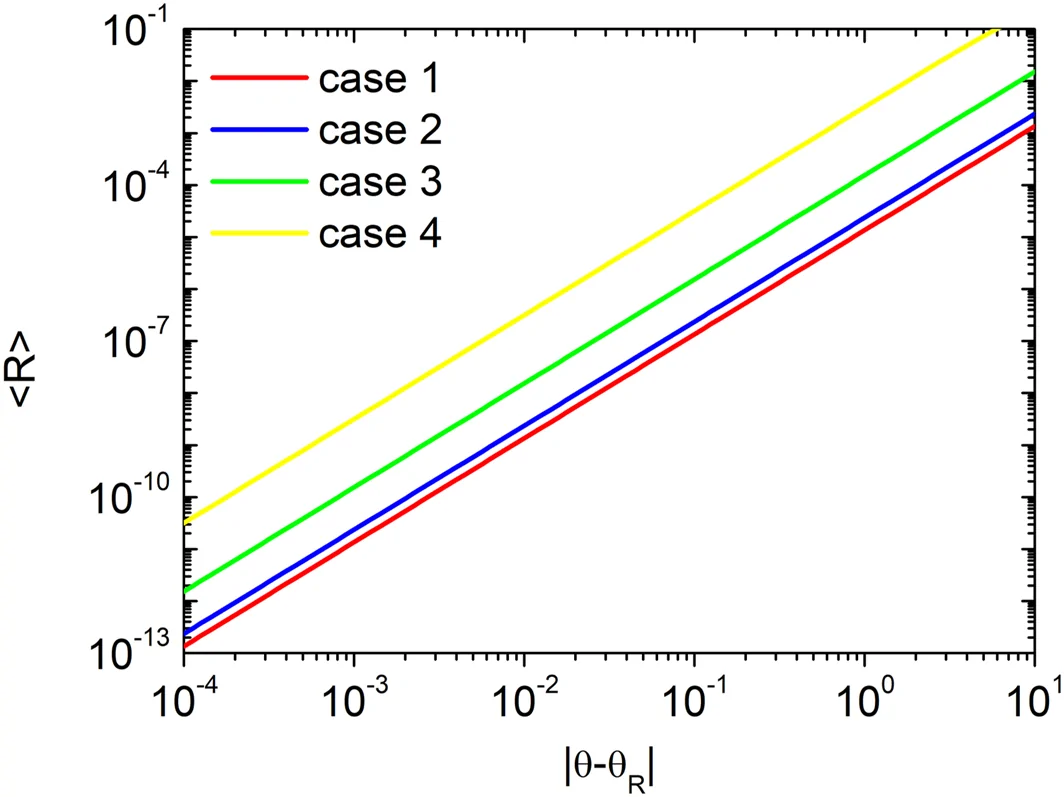
Quadratic scaling of the disorder‐averaged reflectance near the reflectionless directions $\theta =\theta_{R}$ (0 or π). The disorder‐averaged reflectance 〈R〉 is plotted against $\theta -\theta_{R}$ for four representative parameter sets: (1) g=0.01, $\alpha_{0}=0.5$, $\omega_{0}T=10$, and $\theta_{R}=0$; (2) g=0.1, $\alpha_{0}=0.1$, $\omega_{0}T=1$, and $\theta_{R}=0$; (3) g=1, $\alpha_{0}=0.2$, $\omega_{0}T=0.5$, and $\theta_{R}=\pi$; and (4) g=10, $\alpha_{0}=0$, $\omega_{0}T=100$, and $\theta_{R}=\pi$. In all cases, 〈R〉 exhibits the universal quadratic scaling $\langle R\rangle \;\propto \;{\left(\theta -\theta_{R}\right)}^{2}$ in the vicinity of $\theta_{R}$, demonstrating the robustness of the disorder‐enabled directional delocalization against variations in disorder strength g, mean vector potential $\alpha_{0}$, and modulation duration T.

Using the conservation law S+R=1, the quantity 〈S−R〉=1−2〈R〉 represents the net propagation current, proportional to the current density and average group velocity [22]. In the weak disorder regime,

(21) $$\langle S-R\rangle =e^{-t/\tau_{w}},\;\tau_{w}=\frac{1+\alpha_{0}^{2}+2\alpha_{0}\;\cos\;\theta }{2g\omega_{0}\;\sin^{2}\;\theta }.$$

In the strong disorder regime and for large t,

(22) $$\langle S-R\rangle \propto e^{-t/\tau_{s}},\;\tau_{s}=\frac{g}{2\omega_{0}\sin^{2}\;\theta }.$$

Because $g=g_{0}\omega_{0}=g_{0}kv_{F}$, we obtain $\tau_{w}\;\propto \;g_{0}^{-1}k^{-2}$ and $\tau_{s}\;\propto \;g_{0}$, with both scaling as ${\left(\sin\;\theta \right)}^{-2}$. Thus, the net current vanishes asymptotically for all θ≠0,π.

In the weak disorder regime, stronger disorder accelerates the decay of the current. In contrast, in the strong disorder regime, the decay proceeds in two stages: an initial rapid drop to $1-\sin^{2}\;\theta /|\varepsilon_{0}{|}^{2}$ on the timescale ${\left(2g\omega_{0}\right)}^{-1}$, followed by a slower exponential relaxation with a timescale that increases with g. Consequently, sufficiently strong disorder ultimately slows the asymptotic decay of the net current.

Figure 3 presents the temporal evolution of the net current 〈S−R〉 in polar coordinates for various disorder strengths g, with the mean vector potential fixed at $\alpha_{0}=0.5$. The radial coordinate represents time, whereas the angular coordinate corresponds to the incidence angle θ between the wave vector and the vector potential axis. Finite values of 〈S−R〉 indicate sustained propagation, whereas values approaching zero signal localization and suppression of transport.

**FIGURE 3:**
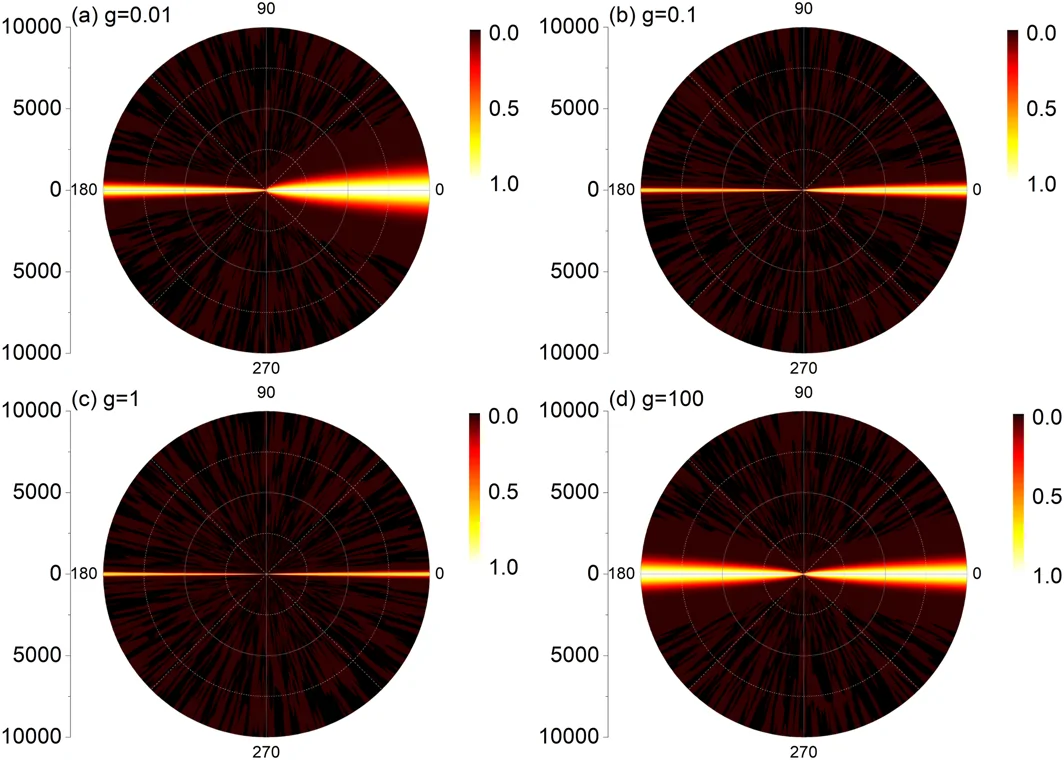
Polar representation of the disorder‐averaged net current 〈S−R〉 under stochastic temporal modulation. The time evolution of 〈S−R〉 is shown in polar coordinates for disorder strengths (a) g=0.01, (b) 0.1, (c) 1, and (d) 100, spanning weak to strong disorder regimes, with fixed mean vector potential amplitude $\alpha_{0}=0.5$. The radial coordinate represents the modulation duration (time), whereas the angular coordinate corresponds to the incidence angle θ. The outermost radius corresponds to the maximum dimensionless duration, $T/\tau_{d}=10^{4}$. Increasing disorder progressively suppresses off‐axis transport, whereas propagation near the reflectionless directions remains robust, demonstrating robust, disorder‐immune directional transport.

As time increases, 〈S−R〉 decays from 1 to 0 for nearly all propagation directions, except within narrow angular regions near θ=0 and π, where transmission persists. These symmetry‐protected directions remain disorder‐immune and define the directional transport channel identified in this work. In the weak disorder regime, the angular window supporting propagation narrows as g increases, whereas in the strong disorder regime, it broadens with further increase of g. In addition, under weak disorder, the decay rate depends asymmetrically on $\alpha_{0}$ and θ [see Equation (21)], leading to directional asymmetry between θ=0 and π, which disappears in the strong disorder limit.

We next examine wave propagation in Model 2. Despite the different statistical structure of the disorder, the qualitative behavior remains unchanged. Figure 4a shows the time evolution of 〈S−R〉 for several incidence angles θ, computed with Δα=1, $\alpha_{0}=0$, and $\omega_{0}\tau_{d}=10$ and averaged over $10^{6}$ realizations. For θ=0, temporal scattering is absent and the net current remains unity, whereas for θ≠0, it decays exponentially, indicating suppression of forward transport due to Anderson localization induced by temporal disorder. Figure 4b shows the disorder‐averaged reflectance 〈R〉 as a function of |θ| for several modulation durations. As in Model 1, 〈R〉 exhibits quadratic scaling near θ=0, $\langle R\rangle \propto \theta^{2}$, confirming the persistence of the disorder‐immune propagation direction.

**FIGURE 4:**
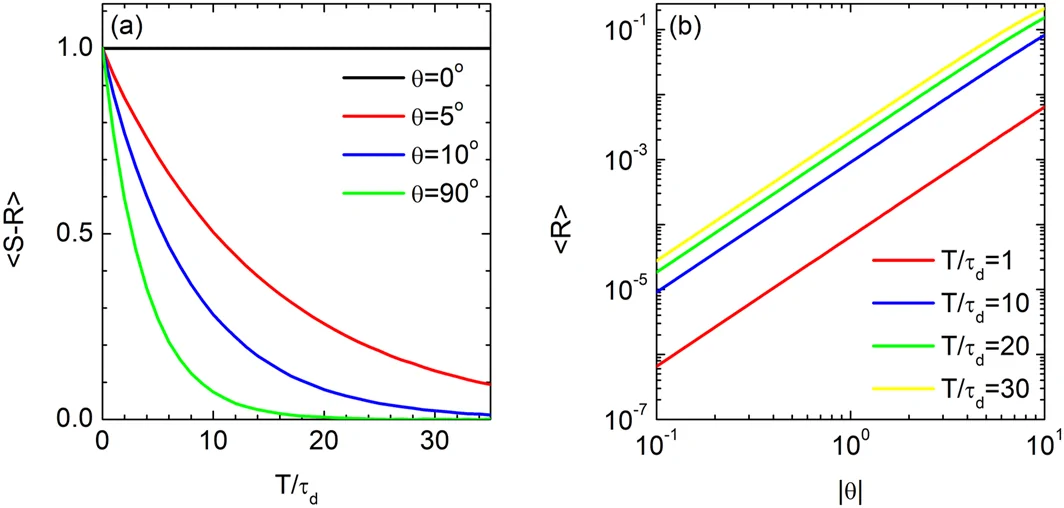
Directional wave localization and reflectance in Model 2. (a) Time evolution of the disorder‐averaged net current 〈S−R〉 for several incidence angles θ, obtained from $10^{6}$ stochastic realizations with dimensionless modulation amplitude Δα=1, mean vector potential amplitude $\alpha_{0}=0$, and dimensionless correlation time $\omega_{0}\tau_{d}=10$, representative of typical modulation conditions. The current remains unity at θ=0, evidencing a disorder‐immune propagation channel, whereas for θ≠0, it decays exponentially in time, indicating Anderson localization induced by temporal disorder. (b) Disorder‐averaged reflectance 〈R〉 versus |θ| for different modulation durations. In the small‐angle regime, $\langle R\rangle \propto \theta^{2}$, demonstrating quadratic suppression near θ=0 and confirming robust directional selectivity.

These results demonstrate that disorder‐induced temporal modulation produces directional localization for all θ≠0,π, independent of the disorder model, while delocalized transport persists only along the symmetry‐protected directions θ=0 and π. This behavior is further illustrated by direct wave packet simulations.

To this end, we consider a two‐dimensional pulse initially centered at the origin with a Gaussian momentum distribution,

(23) $$u\left(x,y,t\right)=\int_{-\infty }^{\infty }\int_{-\infty }^{\infty }D\left(k_{x},k_{y}\right)e^{ik_{x}x+ik_{y}y}\Psi \left(k_{x},k_{y},t\right),dk_{x}dk_{y},$$

where, for t>T,

(24) $$\begin{array}{ll} & \Psi \left(k_{x},k_{y},t\right)=s\left(k_{x},k_{y},T\right)\left(\begin{array}{c} 1\\ \varepsilon_{2}^{*}\left(k_{x},k_{y}\right)/\left|\varepsilon_{2}\left(k_{x},k_{y}\right)\right| \end{array}\right)e^{-i\omega_{2}\left(t-T\right)}\\ & \;\;\;\;\;\;\;\;\;\;\;\;\;\;\;\;+r\left(k_{x},k_{y},T\right)\left(\begin{array}{c} 1\\ -\varepsilon_{2}^{*}\left(k_{x},k_{y}\right)/\left|\varepsilon_{2}\left(k_{x},k_{y}\right)\right| \end{array}\right)e^{i\omega_{2}\left(t-T\right)},\\ & D\left(k_{x},k_{y}\right)=e^{-\frac{k_{x}^{2}+k_{y}^{2}}{2\sigma_{k}^{2}}},\;\omega_{2}=|\varepsilon_{2}|kv_{F}, \end{array}$$

and $\varepsilon_{2}$ is evaluated at the final time corresponding to the modulation duration T. The normalized field intensity is defined as

(25) $$F\left(x,y,T\right)=\frac{|u\left(x,y,T\right){|}^{2}}{\int |u\left(x,y,0\right){|}^{2},dxdy}.$$

Figure 5 illustrates the temporal evolution of a two‐dimensional pulse initially centered at the origin, composed of wave vectors with an isotropic Gaussian distribution. In a stationary medium, the pulse expands uniformly in all directions, forming a circular front whose amplitude decreases due to spatial spreading.

**FIGURE 5:**
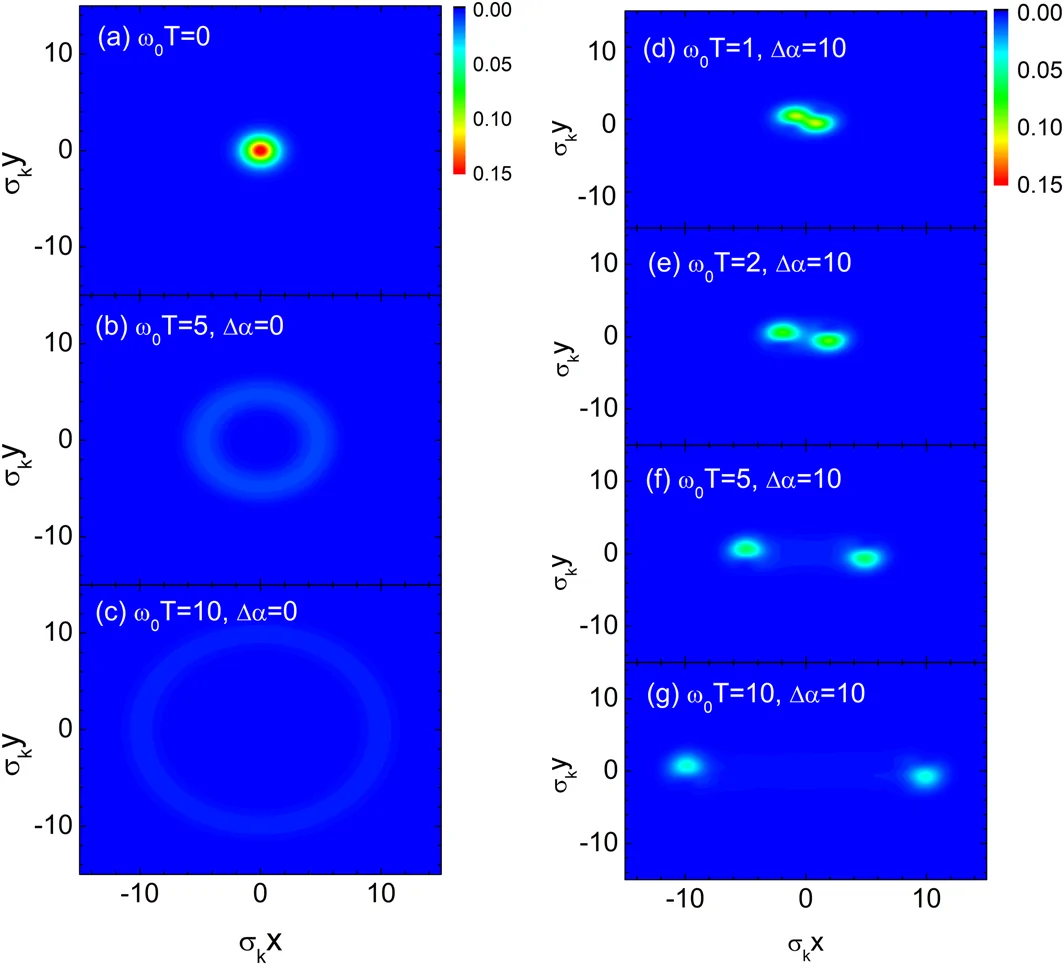
Temporal evolution of a two‐dimensional pulse in stationary and time‐disordered media. (a–c) In a stationary medium, the pulse expands isotropically, spreading uniformly in all directions. (d–g) Under stochastic temporal modulation of the vector potential along the x‐axis with dimensionless modulation amplitude Δα=10, corresponding to a strong modulation regime, the Dirac wave becomes directionally localized and propagates predominantly along the ±x directions, leading to the formation of two symmetric lobes. All results are averaged over 200 disorder realizations with the correlation–time–bandwidth product $v_{F}\sigma_{k}\tau_{d}=1$, for which the disorder memory time is comparable to the inverse spectral bandwidth of the pulse.

When the vector potential undergoes stochastic temporal modulation with strength Δα=10 and fixed orientation along the x‐axis, the Dirac wave becomes directionally localized and propagates only along θ=0 and π. The pulse therefore splits into two components traveling along the ±x directions. This strong directional confinement provides a direct real‐space manifestation of the disorder‐immune transport channel identified above. Although the overall amplitude decreases with time, confinement to two propagation channels substantially slows the decay, allowing the pulse to retain appreciable intensity over extended durations. The resulting two sharply defined propagation lobes demonstrate disorder‐selected transport channels, in which temporal fluctuations actively shape the wave front rather than degrade it.

Figure 6 further demonstrates that the propagation direction can be dynamically controlled in time. In this example, the orientation of the temporally disordered vector potential alternates between the x‐ and y‐axes every $\omega_{0}T=5$, with Δα=10, and results averaged over 200 disorder realizations for $\sigma_{k}\tau_{d}=1$. During the first interval $\left(0<\omega_{0}T<5\right)$, the pulse splits into two lobes along ±x. In the next interval $\left(5<\omega_{0}T<10\right)$, the propagation direction switches to ±y, producing four lobes. When the orientation returns to x $\left(10<\omega_{0}T<15\right)$, the pulse further divides into eight lobes. At $\omega_{0}T=15$, interference near x=0 leads to partial merging, resulting in six distinct lobes.

**FIGURE 6:**
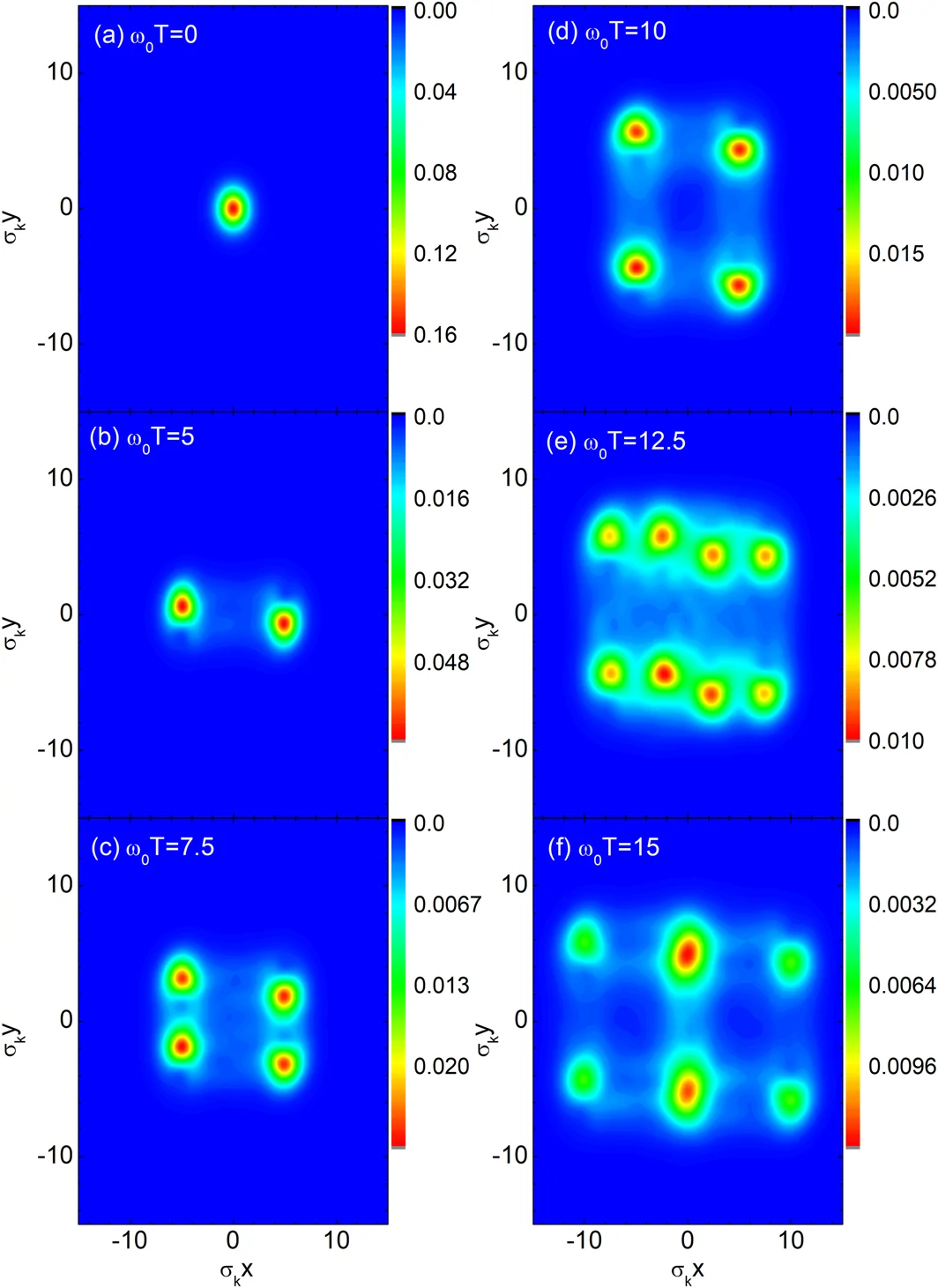
Steering of a Dirac pulse via directional modulation of a time‐disordered vector potential. A Dirac pulse propagates in a medium where the orientation of the temporally disordered vector potential alternates between the x‐ and y‐axes at intervals of $\omega_{0}T=5$, chosen to allow directional lobe formation before switching. The disorder strength is Δα=10, corresponding to a strong modulation regime that enables clear directional splitting. All results are averaged over 200 random realizations with the correlation–time–bandwidth product $v_{F}\sigma_{k}\tau_{d}=1$. (a) The initial Gaussian pulse is centered at the origin. (b) For $0<\omega_{0}T<5$, the vector potential is aligned along x, steering the pulse along ±x and form two lobes. (c and d) For $5<\omega_{0}T<10$, the direction switches to y, redirecting the pulse along ±y and producing four lobes. (e) For $10<\omega_{0}T<15$, the orientation returns to x, further splitting the pulse into eight lobes. (f) At $\omega_{0}T=15$, interference near x=0 leads to partial merging, resulting in six distinct lobes.

These results show that temporal control of the vector potential orientation enables dynamic beam steering and real‐time reconfiguration of transport pathways, providing a direct real‐space manifestation of disorder‐immune directional transport. More broadly, temporal modulation enables programmable routing of wave energy through disorder itself, allowing reconfiguration of propagation pathways without any modification of the underlying spatial structure.

## 5 Discussion

In contrast to conventional wave transport, where disorder typically degrades coherence and suppresses transmission, the present system demonstrates that temporal randomness can instead enforce directional selectivity by dynamically suppressing competing scattering pathways. Temporal disorder thus acts as a resource that isolates a symmetry‐protected transport channel.

This mechanism can be realized in electronic Dirac materials such as graphene, where a time‐dependent vector potential is generated by an in‐plane electric field via E(t)=−∂A(t)/∂t [45]. For representative parameters, taking $v_{F}∼10^{6}$ m/s and a carrier energy of ∼0.1 eV yields a wave number $k\;\approx \;1.5\times 10^{8}$ $m^{-1}$ [46, 47, 48, 49]. The linear Dirac dispersion extends over several hundred meV [48], ensuring the validity of this estimate within the massless regime. For Δα=1 (with α=eA/(ℏk)), the corresponding vector potential variation is $\Delta A\;\approx \;10^{-7}$ V⋅ s/m. Imposing $\omega_{0}\tau_{D}∼10$ then requires an electric field amplitude on the order of $10^{-4}$ V/Å, which lies within demonstrated ultrafast optical capabilities. These parameters remain tunable via carrier energy and modulation bandwidth; at lower carrier energies, the characteristic timescale increases, enabling picosecond‐scale gating while maintaining the condition $\omega_{0}\tau_{D}\approx 10$.

A complementary realization is provided by photonic Dirac systems, such as honeycomb coupled‐resonator (photonic graphene) lattices supporting Dirac cones [50, 51]. In these platforms, synthetic gauge fields can be implemented through dynamic modulation of hopping phases [52]. Electro‐optic phase control in thin‐film lithium niobate and related integrated platforms enables in situ, high‐bandwidth modulation of the coupling phase [53]. Using α=eA/(ℏk), the effective vector potential maps to a Peierls phase Δϕ, yielding Δα∼Δϕ/(qa) near the Dirac point. For typical values qa∼0.1–1, the regime Δα≳1 corresponds to Δϕ∼0.1–1 rad, well within current electro‐optic phase modulation capabilities. Temporal disorder can be implemented via programmable phase sequences, with required modulation speeds compatible with sub‐nanosecond electronics.

The analysis further shows that the disorder‐immune transport channel is fully robust in the massless case, persisting even under random fluctuations of the Fermi velocity. In the presence of a finite mass, the effect is recovered when variations of the vector potential, Fermi velocity, and mass satisfy an appropriate matching condition; otherwise, small mass fluctuations introduce only perturbative corrections (see Supporting Information S1: Section S5).

The underlying mechanism is intrinsically tied to the pseudospin structure of Dirac systems. Interband coupling vanishes identically along specific propagation directions, leading to exact decoupling of transport channels and complete suppression of backscattering. This symmetry‐protected channel persists even under strong temporal disorder and remains robust against parameter fluctuations. By contrast, in conventional electromagnetic systems, random temporal modulation typically induces parametric growth of wave energy at all angles, obscuring any disorder‐selective transparent channel [17, 18, 23]. This highlights a fundamental distinction between Dirac and non‐Dirac wave systems.

These results establish temporal disorder as a controllable design parameter for direction‐selective transport. By exploiting symmetry‐selective dynamics, one can realize functionalities such as dynamic beam steering, adaptive filtering, and reconfigurable routing without relying on spatial structuring. More broadly, this work demonstrates that randomness, when coupled to internal symmetries, can enhance rather than degrade functionality, providing a new paradigm for wave control in time‐modulated systems.

## Author Contributions

**Seulong Kim:** data curation, investigation, formal analysis, writing – original draft, writing – review and editing. **Kihong Kim:** conceptualization, methodology, investigation, formal analysis, funding acquisition, writing – original draft, writing – review and editing.

## Funding

This research was supported by the National Research Foundation of Korea (NRF) grant funded by the Korean Government (Grant RS‐2025‐16071339). It was also supported by the Basic Science Research Program through the NRF, funded by the Ministry of Education (Grant RS‐2021‐NR060141).

## Supporting information


Supporting Information S1

## Data Availability

The data that support the findings of this study are available from the corresponding author upon reasonable request.
