# Supplementary material for: Disorder‐Enabled Directional Delocalization and Wave Steering in Time‐Modulated Dirac Materials
Source: Nanophotonics. 2026 Apr 23;15(9):e70103. doi: 10.1002/nap2.70103 (PMC13642610; doi:10.1002/nap2.70103)
Supplement: Supplementary file 1 [file NAP2-15-e70103-s001.pdf]

Supplementary material for “Disorder-enabled directional delocalization and wave steering in time-modulated Dirac materials”

Seulong Kim[1] and Kihong Kim[2,3*]

[1]Research Institute of Basic Sciences, Ajou University, Suwon, 16499, Korea.
[2]Department of Physics, Ajou University, Suwon, 16499, Korea.
[3]School of Physics, Korea Institute for Advanced Study, Seoul, 02455, Korea.

*Corresponding author(s). E-mail(s): khkim@ajou.ac.kr;

S1. Gauge invariance of temporal scattering

The pseudospin-1/2 Dirac equation with electromagnetic potentials reads

$$i\hbar\frac{\partial}{\partial t}\Psi = v_F\left[\left(\frac{\hbar}{i}\frac{\partial}{\partial x}+eA_x\right)\sigma_x+\left(\frac{\hbar}{i}\frac{\partial}{\partial y}+eA_y\right)\sigma_y\right]\Psi - eV\Psi. \tag{S1}$$

Under a gauge transformation,

$$A \to A+\nabla\Lambda,\ V \to V-\frac{\partial\Lambda}{\partial t}, \tag{S2}$$

and with the transformation of the wave function,

$$\Psi \to \Psi' = e^{i\lambda}\Psi, \tag{S3}$$

the additional terms cancel provided

$$\lambda = -\tfrac{e}{\hbar}\Lambda, \tag{S4}$$

thus ensuring gauge invariance.

Defining the effective wave vector $\mathbf{q} = \mathbf{k} + e\mathbf{A}/\hbar$ through the relation

$$\left(\frac{\hbar}{i}\frac{\partial}{\partial x} + eA_x\right)\Psi = (\hbar k_x + eA_x)\Psi = \hbar q_x \Psi, \tag{S5}$$

one finds that q_x (and, analogously, q_y) is invariant under the gauge transformation. Since the temporal scattering dynamics depend only on the gauge-invariant combination $\epsilon \propto q_x - iq_y$, both the temporal scattering process and the associated directional decoupling condition are gauge-invariant.

S2. Derivation of Eq. (9)

For the time-independent case, assuming a time dependence of $\exp(-i\omega t)$, Eq. (1) can be written as

$$i\hbar\frac{d}{dt}\Psi(t) = \hbar\omega\Psi(t) = \hbar v_F \begin{pmatrix} 0 & k\exp^{-i\theta} + \frac{eA}{\hbar} \\ k\exp^{i\theta} + \frac{eA}{\hbar} & 0 \end{pmatrix}\Psi(t). \tag{S6}$$

This may be rearranged into

$$\begin{pmatrix} \omega & -kv_F\left(\exp^{-i\theta} + \alpha\right) \\ -kv_F\left(\exp^{i\theta} + \alpha\right) & \omega \end{pmatrix}\Psi \equiv M\Psi = 0, \tag{S7}$$

where

$$\alpha = \frac{eA}{\hbar k}. \tag{S8}$$

A nontrivial solution requires $\det(M) = 0$. Evaluating the determinant yields Eq. (9):

$$\omega = \pm k v_F \sqrt{1 + \alpha^2 + 2\alpha\cos\theta}. \tag{S9}$$

Introducing the complex parameter

$$\epsilon = e^{-i\theta} + \alpha, \tag{S10}$$

we obtain

$$\frac{\omega}{\omega_0} = |\epsilon|, \quad \omega_0 \equiv k v_F. \tag{S11}$$

S3. Derivation of Eq. (12)

Consider a temporal interface at $t = 0$, where ϵ changes abruptly from ϵ_1 to ϵ_2. From Eq. (1), the spinor $\Psi(t)$ must remain continuous across the interface. Equation (S6) then gives the relation between ψ_1 and ψ_2:

$$\psi_2 = \frac{i}{k v_F \epsilon}\frac{d\psi_1}{dt}. \tag{S12}$$

For an interface of zero duration ($T = 0$), combination with Eq. (8) gives the boundary conditions

$$\begin{aligned} &s(0) + r(0) = 1, \\ &\frac{\omega_2}{\epsilon_2}\left[s(0) - r(0)\right] = \frac{\omega_1}{\epsilon_1}. \end{aligned} \tag{S13}$$

With $\omega_i = |\epsilon_i|\omega_0$ ($i = 1, 2$), we arrive at Eq. (12):

$$r(0) = \frac{1}{2}\left(1 - \frac{\epsilon_2}{\epsilon_1}\frac{|\epsilon_1|}{|\epsilon_2|}\right), \quad s(0) = \frac{1}{2}\left(1 + \frac{\epsilon_2}{\epsilon_1}\frac{|\epsilon_1|}{|\epsilon_2|}\right). \tag{S14}$$

S4. Proof of the conservation law $S + R = 1$

Reflectance and transmittance are defined as

$$R = |r|^2, \quad S = |s|^2. \tag{S15}$$

From Eq. (10), it follows that

$$\frac{d}{d\tau}(S + R) = s^*\frac{ds}{d\tau} + s\frac{ds^*}{d\tau} + r^*\frac{dr}{d\tau} + r\frac{dr^*}{d\tau} = 0, \tag{S16}$$

since β and γ are real. Therefore, the sum $S + R$ is conserved. At a single interface ($T = 0$), we have

$$\begin{aligned} S(0) + R(0) &= |s(0)|^2 + |r(0)|^2 \\ &= \frac{1}{4}\left(1 + \frac{\epsilon_2}{\epsilon_1}\frac{|\epsilon_1|}{|\epsilon_2|}\right)\left(1 + \frac{\epsilon_2^*}{\epsilon_1^*}\frac{|\epsilon_1|}{|\epsilon_2|}\right) + \frac{1}{4}\left(1 - \frac{\epsilon_2}{\epsilon_1}\frac{|\epsilon_1|}{|\epsilon_2|}\right)\left(1 - \frac{\epsilon_2^*}{\epsilon_1^*}\frac{|\epsilon_1|}{|\epsilon_2|}\right) \\ &= \frac{1}{4}\left(2 + \frac{\epsilon_2}{\epsilon_1}\frac{|\epsilon_1|}{|\epsilon_2|} + \frac{\epsilon_2^*}{\epsilon_1^*}\frac{|\epsilon_1|}{|\epsilon_2|}\right) + \frac{1}{4}\left(2 - \frac{\epsilon_2}{\epsilon_1}\frac{|\epsilon_1|}{|\epsilon_2|} - \frac{\epsilon_2^*}{\epsilon_1^*}\frac{|\epsilon_1|}{|\epsilon_2|}\right) \\ &= 1. \end{aligned} \tag{S17}$$

Hence, $S + R = 1$ always holds.

S5. Effect of Fermi-velocity and mass fluctuations on disorder-enabled directional delocalization

We recall that temporal scattering across an interface (from medium 1 to 2) in pseudospin-1/2 Dirac systems is governed by the parameter

$$f = \frac{\mu_1\mu_2 + v_{x1}v_{x2}q_{x1}q_{x2} + v_{y1}v_{y2}q_{y1}q_{y2}}{\sqrt{\mu_1^2 + v_{x1}^2q_{x1}^2 + v_{y1}^2q_{y1}^2}\sqrt{\mu_2^2 + v_{x2}^2q_{x2}^2 + v_{y2}^2q_{y2}^2}}, \tag{S18}$$

where v_{xi} and v_{yi} are the anisotropic Fermi-velocity components along x and y, $\mu_i = M_i/\hbar$ with M_i the mass energy, and $\mathbf{q} = \mathbf{k} + e\mathbf{A}/\hbar$ [Eq. (20) of Ref. 25]. Directional delocalization arises when $f = \pm 1$, even in the presence of temporal disorder. In this case, scattering corresponds to a total intraband or total interband transition, with no mixing between channels, thereby guaranteeing robust delocalized transport.

For definiteness, let the vector potential be oriented along x ($A_y = 0$). In this case, the directional delocalization condition can be realized only when $k_y = 0$. In the massless case ($\mu_1 = \mu_2 = 0$), one obtains

$$f = \frac{v_{x1}v_{x2}q_{x1}q_{x2}}{\sqrt{v_{x1}^2 q_{x1}^2}\sqrt{v_{x2}^2 q_{x2}^2}} = \mathrm{sgn}(v_{x1}q_{x1})\mathrm{sgn}(v_{x2}q_{x2}) = \pm 1, \tag{S19}$$

showing that the directional delocalization persists exactly, even in the presence of temporal randomness of the Fermi velocity v_x.

For finite μ and $k_y = 0$, the scattering parameter becomes

$$f = \frac{\mu_1\mu_2 + v_{x1}v_{x2}q_{x1}q_{x2}}{\sqrt{\mu_1^2 + v_{x1}^2 q_{x1}^2}\sqrt{\mu_2^2 + v_{x2}^2 q_{x2}^2}}, \tag{S20}$$

which equals ± 1 only if

$$\frac{v_{x1}q_{x1}}{\mu_1} = \frac{v_{x2}q_{x2}}{\mu_2}. \tag{S21}$$

Thus, in massive systems the directional delocalization is not generally realized under arbitrary disorder, but it can still occur when the temporal variations of the vector potential, Fermi velocity, and mass are correlated so as to satisfy Eq. (S21).

If Eq. (S21) is not satisfied, the exact directional delocalization is lost because the additional μ-dependent terms prevent $f = \pm 1$ in general. This is analogous to the massless case at finite k_y, where perfect cancellation is also absent. Nevertheless, for small μ the deviation from $|f| = 1$ is only quadratic:

$$f \approx \mathrm{sgn}(v_{x1}q_{x1})\mathrm{sgn}(v_{x2}q_{x2})\left(1 + \frac{\mu_1\mu_2}{v_{x1}v_{x2}q_{x1}q_{x2}} - \frac{\mu_1^2}{2v_{x1}^2 q_{x1}^2} - \frac{\mu_2^2}{2v_{x2}^2 q_{x2}^2}\right). \tag{S22}$$

Along the directional delocalization direction ($k_y = 0$), this correction is minimized, so scattering remains weakest in that direction.

In summary, although a mass gap generally removes the exact condition for directional delocalization, the suppression of backscattering remains nearly intact for moderate masses. As a result, transport retains the essential features of the massless Dirac limit, with only perturbative deviations.
